# A common registration-to-publication automated pipeline for nomenclatural acts for higher plants (International Plant Names Index, IPNI), fungi (Index Fungorum, MycoBank) and animals (ZooBank)

**DOI:** 10.3897/zookeys.550.9551

**Published:** 2016-01-07

**Authors:** Lyubomir Penev, Alan Paton, Nicola Nicolson, Paul Kirk, Richard L. Pyle, Robert Whitton, Teodor Georgiev, Christine Barker, Christopher Hopkins, Vincent Robert, Jordan Biserkov, Pavel Stoev

**Affiliations:** 1 Institute for Biodiversity and Ecosystem Research, Bulgarian Academy of Sciences, Sofia, Bulgaria; 2 Pensoft Publishers, Sofia, Bulgaria; 3 National Museum of Natural History, Sofia, Bulgaria; 4 International Plant Name Index (IPNI) and Index Fungorum (IF), Royal Botanic Gardens Kew, UK; 5 Mycobank, CBS Fungal Biodiversity Centre, Utrecht, The Netherlands; 6 ZooBank, Bishop Museum, Honolulu, USA

**Keywords:** Taxon names, nomenclatural acts, pre-publication registration, International Plant Name Index (IPNI), Index Fungorum, Mycobank, ZooBank

## Abstract

Collaborative effort among four lead indexes of taxon names and nomenclatural acts (International Plant Name Index (IPNI), Index Fungorum, MycoBank and ZooBank) and the journals PhytoKeys, MycoKeys and ZooKeys to create an automated, pre-publication, registration workflow, based on a server-to-server, XML request/response model. The registration model for ZooBank uses the TaxPub schema, which is an extension to the Journal Tag Publishing Suite (JATS) of the National Library of Medicine (NLM). The indexing or registration model of IPNI and Index Fungorum will use the Taxonomic Concept Transfer Schema (TCS) as a basic standard for the workflow. Other journals and publishers who intend to implement automated, pre-publication, registration of taxon names and nomenclatural acts can also use the open sample XML formats and links to schemas and relevant information published in the paper.

International Plant Name Index

Journal Tag Publishing Suite

Taxonomic Concept Transfer Schema

## Introduction

The process of indexing nomenclatural acts from published literature has a long tradition, in some cases dating as far back as the middle of 19^th^ century for different taxonomic groups. As a result there are several nomenclatural indexes that aim to be comprehensive for their focal taxa, for example, Index Kewensis in botany, Index Fungorum or MycoBank in mycology, and Zoological Record and *Index Animalium* in zoology. Sherborn’s effort in *Index Animalium* surely stands as the giant among these efforts due to the sheer scale of described animal diversity (Evenhuis 2016; Miller 2016; Taylor 2016; Dickinson 2016; Pilsk et al. 2016; Welter-Schultes et al. 2016). Taxonomists use these indexes to trace nomenclatural acts through the literature and to help ensure they have considered relevant published works. They can also, along with a far broader audience, use them as an authoritative source of information including the correct spelling and authorship of the name. This improves information retrieval and fidelity (Lyal 2016; Page 2016; Pyle 2016). Increasingly these indexes are being used as the basis for a Global Names Architecture (www.globalnames.org, Patterson et al. 2010). Names from these indexes are either used directly in, or could be linked to, almost all information resources which contain information about organisms. Names also form the foundation for taxonomic concepts, which could be considered as a set of related names. Thus, these indexes occupy a vital role in connecting the occurrence of names in literature and diverse information systems with differing taxonomic concepts (Patterson et al. 2010).

Historically, these indexes have been compiled by a team of editors scanning the relevant literature. This is an inefficient process. The lists become outdated even as they are being produced, because newly described taxa are continually being added to the list. However, the increase in electronic publication of nomenclatural acts made possible by recent changes to the nomenclatural codes in zoology (ICZN 2012) and botany (Knapp et al. 2011, ICNafp 2012) provides an opportunity to reduce the time spent by index editors on keying information already processed by the author and publisher. By reducing the scanning times and manual compilation, index editors would be freed to spend more time ensuring the data quality of the index, facilitating greater linkages with other information resources. Therefore, the indexes should switch focus from *post-publication indexing* to *pre-publication registration*.

Electronic registration of nomenclatural acts in trusted online registries would have the advantage of ensuring nomenclatural novelties published according to the relevant code would be broadly disseminated and available for linkage into other systems. Registration needs to be developed in accordance with the revisions of the biological codes of nomenclature to make the most efficient use of developing web technologies. Mandatory registration would ensure that all new nomenclatural acts governed under the code were captured and treated consistently (Polaszek et al. 2005). However, if such a system were to be open, and work with broadly agreed standards, it could also open up the indexing and registration process to a broader range of actors, thus improving the scope and speed of data capture within the indexes, the linkage between indexes, and facilitating the creation of new indexes or registries which could complement or cover gaps between existing resources.

This paper deals with a specific and important part of the registration process, namely a common model for an automated, prior to publication, machine-to-machine, XML-based registration and associated workflow between publishers and indexes who could act as registries in further streamlining the process of registration and making it cost efficient.

## Current status

There are several ways as to how registration (or indexing if registration is not yet mandated by the relevant code) can be best implemented. Different options and the relationship to the publication process have been extensively reviewed by Pyle and Michel (2008) and Morris et al. (2011). The concept of an automated registration model was first presented by several of the authors of this article at the Sherborn meeting in London in October 2011 and at the Biosystematics 2013 Conference in Vienna, in February 2013, with an expansion at the Digital Nomenclature Workshop in London in January 2015.

Despite the visible progress in recent years, four major questions remain to be answered:

1. When exactly should the registration of a nomenclatural act take place – before or after publication?
2. Who should be responsible for the registration of the act – authors, registry curators or publishers?
3. How is registration actually effected?
4. Who validates the accuracy of the bibliographic metadata for any registered act?

The International Botanical Congress in Melbourne in July 2011 had a major impact on streamlining the process by amending the International Code of Nomenclature for algae, fungi, and plants (ICNafp) such that, from 1 January 2013 to be validly published all new names of fungi must be registered before publication and identifiers for each name included in the publication (Miller et al. 2011, Knapp et al. 2011, McNeill et al. 2012, see also ICNafp 2012).

Shortly thereafter, The International Commission on Zoological Nomenclature voted in favour of a revised version of the amendment to the International Code of Zoological Nomenclature that was first proposed in 2008. The purpose of the amendment is to expand and refine the methods of publication allowed by the Code, particularly in relation to electronic publication. The amendment establishes an Official Register of Zoological Nomenclature (with ZooBank as its online version), allows electronic publication after 2011 under certain conditions, and disallows publication on optical discs after 2012. The requirements for electronic publications are that the work be registered in ZooBank before it is published, that the work itself states the date of publication and contains evidence that registration has occurred, and that the ZooBank registration states both the name of an electronic archive intended to preserve the work and the ISSN or ISBN associated with the work. Registration of new scientific names and nomenclatural acts is not required. The Commission confirmed that ZooBank was ready to handle the requirements of the amendment [International Commission on Zoological Nomenclature (ICZN) 2012].

The current situation with indexing and registration in the three domains of eukaryotic organisms can be summarized as follows:

**FUNGI**

- Post-publication Indexing in Index Fungorum (IF) and MycoBank (MB)
- Pre-publication registration mandatory for fungi since 1^st^ of January 2013
- Record identifiers must be published in the protologue
- Three official registries are approved: MycoBank, Index Fungorum, Fungal Names

**PLANTS**

- Post-publication indexing is a well-established practice of the International Plant Names Index (IPNI) which covers seed plants, ferns and lycophytes but not bryophytes or algae
- Pre-publication indexing and inclusion of IPNI record identifiers in protologues piloted with Phytokeys, PLoS ONE and Kew Bulletin

**ANIMALS**

- Post-publication indexing is a well-established practice of Zoological Record (now published by Thomson Reuters)
- Pre-publication registration in ZooBank mandatory since 1^st^ of January 2012 for e-only publications
- Record identifiers should be published in the original description

Registration of many new nomenclatural acts might be a tedious and extremely time-consuming process if done “by hand”, especially in the recently introduced but increasingly submitted “turbo-taxonomic” papers, combining molecular data, concise morphological descriptions and digital imaging (Butcher et al. 2012, Riedel et al. 2013). The numbers of new taxa described in such papers may count in hundreds, for example 178 new species of parasitic wasps (Butcher et al. 2012) and 101 new species of *Trigonopterus* weevils (Riedel et al. 2013). The ultimate record is held by the paper of Marsh et al. (2013) describing 277 new braconid wasps from Costa Rica. This paper is remarkable also because it became the first “turbo-taxonomic” paper where all 277 new species were registered in Zoobank automatically in just a few seconds, saving a great deal of time to the authors, publisher and the registry.

## Which nomenclatural acts are subject of registration?

There are significant differences in the scope and number of nomenclatural acts that are tracked by the current indexes and registries (Table 1). In several cases, acts are treated differently by the biological codes. For example, new suprafamilial names and new combinations are governed by the ICNafp, but not by ICZN.

**Table 1.** Nomenclatural acts that are recorded by the indexing services and could potentially be a subject of pre-publication registration in botany, mycology and zoology.

Taxonomic / nomenclatural act	IPNI (botany: vascular plants)	Index Fungorum (mycology)	MycoBank (mycology)	ZooBank (zoology)
New taxon:				
- suprafamilial	-	+	+	
- familial	+	+	+	+
- infrafamilial	+	+	+	+
- generic	+	+	+	+
- infrageneric	+	+	+	+
- specific	+	+	+	+
- infraspecific	+	+	+	+
- hybrids^1^	+	+	+	n/a
New replacement name	+	+	+	
New combination	+	+	+	
Tautonym^2^	+	+	+	n/a
Typifications^3^				
- holotype	+	+	+	
- lectotype	-	+	+	
- neotype	-	+	+	
- epitype	-	+	+	n/a

1 Hybrids need not be treated as a category of new taxon, but there needs to be a mechanism of flagging the ranks above as hybrids where necessary

2 Tautonyms are not validly published in ICNafp. IPNI (and IF) record tautonyms if published, but such cases should be picked up at the indexing stage.

3 IPNI normally does not record new lectotypifications, but does include typifications of new taxa at generic rank and below.

## The registration workflow

In our view, the registration (or indexing in groups where registration is not yet mandated by the code) of nomenclatural acts and the quality control of the bibliographic metadata in these registries should be a primary responsibility of publishers and registry curators and, to a lesser extent, of authors. Registration of a nomenclatural act could be initiated by an author, at the pre-submission or pre-acceptance for publication stage. However, we prefer the publisher-initiated model as it avoids registry curators curating data that may never be published according to the rules of the relevant code. Such a practice may lead to “over-saturation” of the registries with names that are not validly published, causing confusion. Focusing on names accepted for publication also allows these curators more time to focus on the published act and this may allow these specialist staff to assist publication by identifying inconsistencies with the relevant code. Moreover, the publishers’ role is essential in checking and correcting the pre-publication registration details against the ultimately published information. The model presented below could easily be adapted for author initiation, though we envisage that there would be a greater curatorial overhead and a greater likelihood of errors being created. However, we accept that the model needs to be flexible and allow alternatives if it is to receive community support.

**Figure 1.**
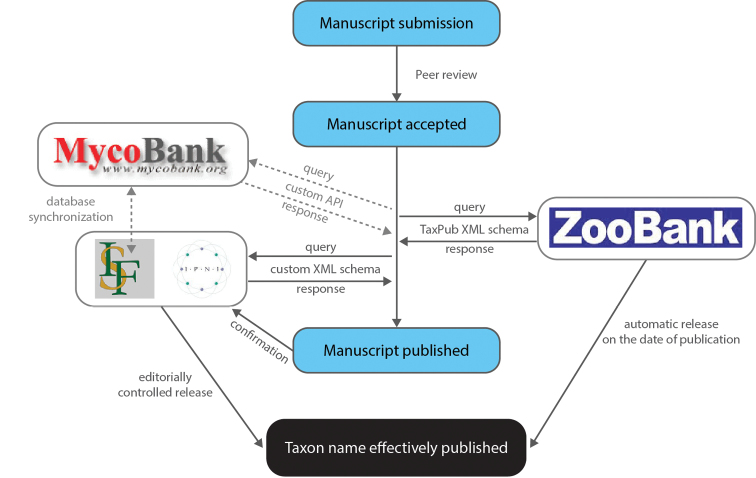
Automated registration process and validation of finally published data and metadata between publisher and registry. Abbreviation on logos: IPNI - International Plant Name Index, IF - Index Fungorum.

In the “journal-centric” model, the registration of taxonomic and nomenclatural acts involves two main classes of actors: (1) publishers, or editors, and (2) registry curators. The publisher takes the responsibility for initiating the registration of nomenclatural acts so that the workflow can be performed following a common stepwise model (see also Fig. 1):

Step 1. XML message from the publisher to the registry on acceptance of the manuscript containing the type of act, taxon names, and preliminary bibliographic metadata; the registry will store the data *but not make these publicly available before the final publication date*.

Step 2a. Response XML report containing the unique identifier of the act as supplied by the registry and/or any relevant error messages.

Step 2b. Error correction and de-duplication performed manually: human intervention, at either registry’s or publisher’s side (or at both).

Step 3. Inclusion of registry supplied identifiers in the published treatments (protologues, nomenclatural acts).

Step 4. Making the information in the registry publicly accessible upon publication, providing a link from the registry record to the article.

The registration process should be as automated as possible. There are several reasons to maximize automation of registration, the most significant being:

- Increasing cases of bulk, “turbo-taxonomic”, descriptions of new taxa within a single paper, sometimes counted in hundreds, which creates significant overhead on the authoring and editorial process.
- Decreased risk of errors caused by human intervention (e.g. re-typing).
- Disambiguation of the dates of acceptance and publication of a manuscript.
- Efficient and accurate validation of final published data and metadata through automated export from the publisher to the registry on the day of publication.

## The automated registration process

Within the framework of the EU FP7 project pro-iBiosphere, and in close collaboration with Zoological Record, ZooBank, IPNI, MycoBank and Index Fungorum, as well as with the Global Names project (www.globalnames.org) we are developing a workflow and associated XML formats to streamline the registration of nomenclatural acts within the pre-publication process. The workflow was piloted by IPNI for higher plants and ZooBank for animals and the journals PhytoKeys and ZooKeys, respectively. The formats differ between the two main biological codes, ICNafp and ICZN, hence we describe these separately below.

### Automated indexing with the International Plant Name Index (IPNI)

The pre-publication indexing of new plant taxa and nomenclatural acts in IPNI and inclusion of the IPNI identifiers in the protologues was first trialled in the journal PhytoKeys since the publication of its first issue in 2010 (Penev et al. 2010). With the pro-iBiosphere project the workflow has been piloted to include an automated registration module. The pilot project uses a custom XML format illustrated by a new genus *Lettowia* description and new combination *Lettowia nyassae* (Oliv.) H. Rob., comb. nov. in the paper of Robinson and Skvarla (2013) (Appendices 1 and 2). The emphasis of the pilot was to understand the workflow; as this is scaled up to production use with a broader range of partners, IPNI will move to use the Taxon Concept Schema standard to encode the data exchanged. This will enable broader adoption.

The XML query is submitted to IPNI’s Application Programming Interface (API) through a POST request and replied back with automatically inserted IPNI identifiers.

### Automated registration with Index Fungorum

The registration workflow of Index Fungorum (IF) will adopt that of IPNI after the IF system has moved to Royal Botanic Gardens Kew to run alongside IPNI.

### Automated registration with MycoBank

The following methods of the MycoBank API are enough for a straightforward implementation:

1. SearchMycoBankWithFilters
2. InsertUserProfile
3. UpdateUserProfile
4. InsertMycobankRecord
5. UpdateMycobankRecord

Using the combinations (1, 2, 3) and (1, 4, 5) one can implement the Upsert (Update if exists, Insert otherwise) semantics required for the the Common query/response registration model.

As there are multiple fungi registries (MycoBank, Index Fungorum, Fungal Names), another approach would be to perform the registration with only one of them and rely on the synchronization mechanisms (currently being built) to propagate the information to the other databases.

### Automated registration with ZooBank

Similarly to the case of PhytoKeys, ZooKeys was the first journal that implemented a mandatory registration of new taxon names in zoology, since the publication of its first issue in 2008 (Penev et al. 2008). The automated registration with ZooBank is based on a slightly different approach than that with IPNI and uses the TaxPub XML schema (Catapano 2010) as a basic standard. Upon acceptance and producing the XML version of the manuscript, we upload it on the ZooBank server through the ZooBank’s interface (see Suppl. material 3 for the submitted TaxPub XML format). Then a software tool at ZooBank harvests the TaxPub XML and registers the title, authors and new taxon names. The tool also checks if some or all authors have been previously registered and inserts their current (or newly registered) ZooBank UUIDs. In case in the ZooBank database there are authors with identical names (homonyms), the interface displays these so that the operator at the editorial office could disambiguate the overlapping authors’ names by selecting the right one. The whole TaxPub XML is sent back with inserted UUIDs for the article, authors and new names (Suppl. material 4). In case the manuscript XML has been changed after the registration process, it can be uploaded again and the new data will replace the previous ones. At the day of publication, the names and the bibliographic metadata are made publicly available in ZooBank.

## What other journal publishers should do to use the workflow?

The registration workflow and XML formats published in this article are free to use for anyone who would like to implement it. To ensure broader adoption of the registration model, the data exchanged through the workflow should be encoded in a standard. For zoology, journals should adopt the TaxPub XML schema (Catapano 2010; open source available at: https://github.com/tcatapano/TaxPub/releases/tag/v0.5-beta) which encodes publications as required by the zoological code. For botany, the registration workflow uses currently a custom XML format based on the Taxon Concept Transfer Schema (TCS) XML schema (http://www.tdwg.org/standards/117/) which encodes names; in the future, IPNI and Index Fungorum will implement TCS as a basic standard for registration/indexing of new names and other nomenclatural acts.

The Suppl. materials 1–4 show some data encoded for the pilot project using a custom XML format – whilst this shows the kind of data that will be exchanged, it should not be used as a template – the TCS and TaxPub standards should be used as reference.

Once the editorial workflow is defined, and structured data can be produced according to these standards, journal editors should contact registries for access to their Application Programming Interfaces (APIs).

The authors of this article, staff at the registries and at Pensoft are available to consult journals who intend to implement the automated registration process. Future changes to the automated registration workflow will be published on the Wiki page of the pro-iBiosphere project at http://wiki.pro-ibiosphere.eu/wiki/Pilot_2.

## Supplementary materials

Supplementary material 1 XML response of IPNI Data type: (measurement/occurence/multimedia/etc.) Explanation note: XML query sent from Pensoft to IPNI on the day of acceptance of the manuscript for publication [exemplified with the paper of Robinson and Skvarla (2013)]. This dataset is made available under the Open Database License (http://opendatacommons.org/licenses/odbl/1.0/). The Open Database License (ODbL) is a license agreement intended to allow users to freely share, modify, and use this Dataset while maintaining this same freedom for others, provided that the original source and author(s) are credited.Lyubomir Penev, Alan Paton, Nicky Nicolson, Paul Kirk, Richard Pyle, Robert Whitton, Teodor Georgiev, Christine Barker, Christopher Hopkins, Vincent Robert, Jordan Biserkov, Pavel Stoev

Supplementary material 2
IPNI response XML Data type: (measurement/occurence/multimedia/etc.) Explanation note: XML response of IPNI to the query in Suppl. material 1. The response is sent back to Pensoft and contains the registration numbers of the new genus name and the new combination [exemplified with the paper of Robinson and Skvarla (2013)]. This dataset is made available under the Open Database License (http://opendatacommons.org/licenses/odbl/1.0/). The Open Database License (ODbL) is a license agreement intended to allow users to freely share, modify, and use this Dataset while maintaining this same freedom for others, provided that the original source and author(s) are credited.Lyubomir Penev, Alan Paton, Nicky Nicolson, Paul Kirk, Richard Pyle, Robert Whitton, Teodor Georgiev, Christine Barker, Christopher Hopkins, Vincent Robert, Jordan Biserkov, Pavel Stoev

Supplementary material 3 XML response of TaxPub Data type: (measurement/occurence/multimedia/etc.) Explanation note: TaxPub XML of a ready-to-publish manuscript submitted from Pensoft to ZooBank [exemplified with the paper of Morffe and Rodríguez (2013)]. This dataset is made available under the Open Database License (http://opendatacommons.org/licenses/odbl/1.0/). The Open Database License (ODbL) is a license agreement intended to allow users to freely share, modify, and use this Dataset while maintaining this same freedom for others, provided that the original source and author(s) are credited.Lyubomir Penev, Alan Paton, Nicky Nicolson, Paul Kirk, Richard Pyle, Robert Whitton, Teodor Georgiev, Christine Barker, Christopher Hopkins, Vincent Robert, Jordan Biserkov, Pavel Stoev

Supplementary material 4 TaxPub response XML Data type: (measurement/occurence/multimedia/etc.) Explanation note: TaxPub XML returned from ZooBank to Pensoft containing UUIDs of the article, authors and new taxon names [exemplified with the paper of Morffe and Rodríguez (2013)]. This dataset is made available under the Open Database License (http://opendatacommons.org/licenses/odbl/1.0/). The Open Database License (ODbL) is a license agreement intended to allow users to freely share, modify, and use this Dataset while maintaining this same freedom for others, provided that the original source and author(s) are credited.Lyubomir Penev, Alan Paton, Nicky Nicolson, Paul Kirk, Richard Pyle, Robert Whitton, Teodor Georgiev, Christine Barker, Christopher Hopkins, Vincent Robert, Jordan Biserkov, Pavel Stoev
